# Case Report: Hereditary angioedema masquerading as gastroenteritis

**DOI:** 10.3389/fimmu.2026.1828352

**Published:** 2026-08-26

**Authors:** Xiaofeng Ren, Jialin Wu, Yajun Xu

**Affiliations:** 1 Department of Gastroenterology, Sanmenxia Central Hospital, Sanmenxia, China; 2 Department of Gastroenterology, Beijing Friendship Hospital, Capital Medical University, Beijing, China; 3 Department of Radiology, Henan University of Science and Technology Affiliated Yellow River Hospital, Sanmenxia, China

**Keywords:** abdominal attacks, C1-inhibitor deficiency, cascade screening, diagnostic delay, HAE-C1INH, hereditary angioedema, SERPING1

## Abstract

Hereditary angioedema with C1-inhibitor deficiency (HAE-C1INH) is a rare bradykinin-mediated disorder that may present predominantly with gastrointestinal symptoms, leading to diagnostic delay and unnecessary interventions. We report a 26-year-old man with recurrent abdominal pain, watery diarrhea, bowel wall edema, and transient ascites since 2022, repeatedly misdiagnosed as infectious gastroenteritis. He also reported recurrent hand and foot swelling. Complement testing showed low C4 and reduced C1-inhibitor function, and repeat testing confirmed low C1-inhibitor antigen and function, supporting type 1 HAE-C1INH. During follow-up, genetic testing identified a heterozygous likely pathogenic nonsense variant in SERPING1, NM_000062.3:c.1480C>T (p.Arg494Ter/R494*). Family investigation of 50 relatives identified 13 clinically suspected affected individuals, including three who reportedly died from laryngeal edema; laboratory confirmation was available for the proband and two relatives. This case emphasizes that recurrent, self-limited abdominal attacks with transient ascites should prompt C4 and C1-inhibitor testing and family counseling.

## Introduction

Hereditary angioedema (HAE) is a rare, potentially life-threatening disorder characterized by recurrent, non-pruritic angioedema involving the skin, gastrointestinal tract, and upper airway. Current classification distinguishes HAE with C1-inhibitor deficiency (HAE-C1INH), including type 1 and type 2 disease, from HAE with normal C1-inhibitor (HAE-nC1INH), for which several genetically defined subtypes have been described, including variants in F12, PLG, ANGPT1, KNG1, MYOF, HS3ST6, CPN1, and DAB2IP (1, 2). Type 1 HAE-C1INH is characterized by low antigenic and functional C1-inhibitor (C1-INH), whereas type 2 HAE-C1INH usually shows normal or elevated antigenic C1-INH with reduced function.

In HAE-C1INH, SERPING1 variants lead to quantitative or functional deficiency of C1-INH. Insufficient regulation of the plasma contact system results in excessive bradykinin generation, increased vascular permeability, and recurrent subcutaneous or submucosal edema (3). Gastrointestinal involvement is common and may manifest as severe abdominal pain, vomiting, watery diarrhea, bowel wall edema, and sterile ascites (4, 5). Because these attacks are transient and may resolve spontaneously, abdominal HAE can be misdiagnosed as infectious gastroenteritis, inflammatory bowel disease, appendicitis, or other causes of acute abdomen.

We describe a young man with recurrent abdominal attacks initially attributed to gastroenteritis, in whom complement testing, family investigation, and a newly obtained SERPING1 genetic result supported the diagnosis of type 1 HAE-C1INH. The case also illustrates the need for cautious interpretation of pedigree-based screening when laboratory or genetic confirmation is incomplete.

## Case report

A 26-year-old man was admitted to the gastroenterology unit in July 2025 with a 3-day history of periumbilical colicky pain and more than 8 episodes of watery diarrhea per day. He had experienced similar episodes in 2022, 2023, and January 2025; each resolved spontaneously within 48–72 hours and had been diagnosed as ‘acute gastroenteritis’.

The patient also reported recurrent swelling of the hands and feet. He could not precisely recall the first occurrence of these peripheral swelling episodes, but they were considered to have begun approximately around the time of his first abdominal episode in 2022. He denied previous facial, tongue, or laryngeal swelling, and no genital attack was reported. Thus, 2022 was regarded as the first clearly documented onset. He reported no fever, vomiting or gastrointestinal bleeding.

**Figure 1:**
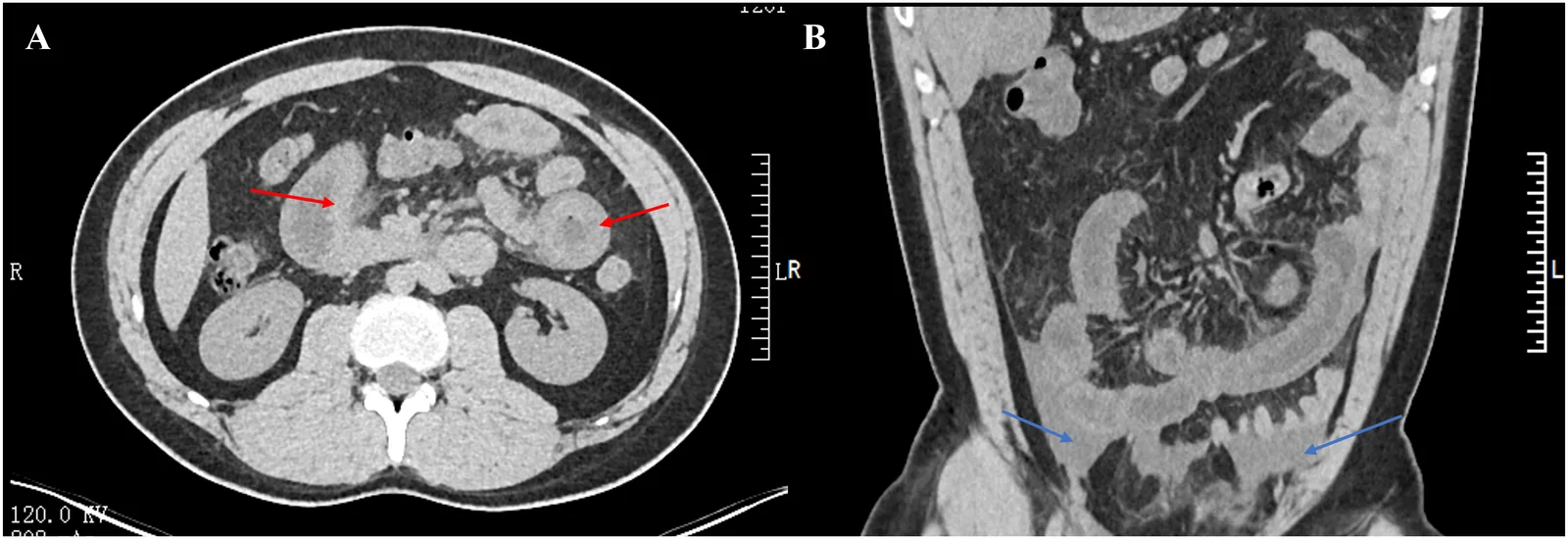
Computed tomography showing circumferential thickening of the duodenum and proximal jejunum (red arrows) with moderate ascites (blue arrows).

Physical examination revealed stable vital signs, diffuse abdominal tenderness, and shifting dullness. Laboratory tests showed leukocytosis (white blood cell count 11.4 x 10^9^/L, 83% neutrophils), elevated C-reactive protein (33 mg/L), normal albumin (42.9 g/L), and normal pancreatic enzymes. Computed tomography revealed circumferential wall thickening of the duodenum and proximal jejunum with peri-enteric inflammation and moderate ascites (maximum depth 41 mm), suggesting small-bowel edema rather than a fixed obstructive lesion (**Figure 1**).

Diagnostic paracentesis yielded sterile, clear fluid (protein 38 g/L, cell count 180/µL, 70% lymphocytes). Stool cultures, Clostridioides difficile toxin assay, and fecal calprotectin were negative. Empirical treatment with amoxicillin, levofloxacin, and methylprednisolone before admission had been ineffective. After admission, cefoperazone-sulbactam was initiated as empirical anti-infective therapy. On hospital day 4, the abdominal pain resolved abruptly, and repeat ultrasonography showed complete resolution of ascites. The diagnostic course during the index episode and subsequent follow-up are summarized in **Table 1**.

**Table 1:** Timeline of the episode of care and follow-up.

Time point	Clinical events
2022	First clearly documented episode of abdominal pain and watery diarrhea, diagnosed as gastroenteritis; recurrent hand/foot swelling was retrospectively considered to have begun around the same period.
2023	Second self-limited episode with similar abdominal symptoms, again diagnosed as gastroenteritis.
January 2025	Third abdominal episode, treated with antibiotics without clear benefit.
July 2025, Day 0	Admission with periumbilical pain, watery diarrhea, ascites; CT showed duodenal/proximal jejunal wall thickening and moderate ascites
Day 1-3	Paracentesis showed sterile clear fluid; stool culture, Clostridioides difficile toxin assay, and fecal calprotectin were negative. In-house complement/C1-INH testing showed low C4 and reduced functional C1-INH activity.
Day 4-5	Abdominal pain resolved abruptly; repeat ultrasound showed resolution of ascites; external reference laboratory testing showed low C1-INH antigen, low C1-INH function, and C4 at the lower end of the reference range.
After diagnosis	A single subcutaneous dose of icatibant 30 mg was administered in the setting of the recently resolving acute abdominal edema episode; counseling was provided.
Post-discharge follow-up	No recurrence of abdominal pain, diarrhea, peripheral swelling, or upper-airway symptoms was reported. Genetic testing identified a heterozygous likely pathogenic SERPING1 nonsense variant.

The recurrent, self-limited nature of the episodes, transient ascites, bowel wall edema, negative microbiological evaluation, limited response to antibiotics, and abrupt spontaneous resolution prompted reconsideration of the diagnosis. HAE-C1INH was suspected because transient ascites with bowel wall edema is a recognized feature of abdominal HAE attacks (5).

Two sets of complement and C1-INH measurements were obtained during the index hospitalization. On hospital day 2, in-house testing showed a markedly reduced serum C4 level of 0.05 g/L (reference range 0.10–0.40 g/L), antigenic C1-INH of 0.22 g/L (reference range 0.21–0.39 g/L), and reduced functional C1-INH activity of 22% of normal. Repeat testing performed by an external reference laboratory on hospital day 4 showed low C1-INH antigen and markedly reduced C1-INH function, with C4 at the lower end of the external reference range, further supporting the diagnosis of type 1 HAE-C1INH (**Table 2**).

**Table 2:** Complement phenotype of the proband and laboratory-confirmed relatives.

Individual/time point	Symptoms	Laboratory/sample	C1-INH antigen	fC1-INH	C4
Proband/Patient 1 (P1), hospital day 2	Abdominal pain, diarrhea, ascites; history of hand/foot swelling	In-house laboratory	0.22 g/L (reference 0.21–0.39)	22% of normal	0.05 g/L (reference 0.10–0.40)
Proband, Patient 1 (P1), hospital day 4	Same index episode	External reference laboratory	22.47 µg/mL (reference 81.46–291.29)	<7% (reference ≥58.9%)	73.48 µg/mL (reference 72.85–372.95)
Patient 2 (P2), proband’s father	Recurrent swelling of hands, feet, and scrotum	External reference laboratory	11.63 µg/mL (reference 81.46–291.29)	11.18% (reference ≥58.9%)	61.67 µg/mL (reference 72.85–372.95)
Patient 3 (P3), paternal second cousin	Recurrent abdominal pain	External reference laboratory	8.93 µg/mL (reference 81.46–291.29)	9.47% (reference ≥58.9%)	21.05 µg/mL (reference 72.85–372.95)

After the diagnosis was established, the patient received subcutaneous icatibant 30 mg in the setting of the recently resolving acute abdominal edema episode because short-term recrudescence or progression remained a concern. No immediate adverse reaction was observed. The patient was counseled regarding avoidance of unnecessary antibiotics, recognition of laryngeal symptoms, and the need for urgent medical attention during airway attacks. During follow-up from July 2025 to the revision period, he reported no recurrent abdominal pain, diarrhea, peripheral swelling, or upper airway symptoms, and no additional icatibant dose was required.

During post-discharge follow-up, genetic testing of the proband was completed. A heterozygous nonsense variant in SERPING1, NM_000062.3:c.1480C>T (p.Arg494Ter/R494*), was identified and classified as likely pathogenic by the clinical genetic testing report (**Table 3**). This molecular finding, together with the complement profile and compatible phenotype, further supported the diagnosis of SERPING1-associated type 1 HAE-C1INH. This variant has previously been reported in Chinese patients with HAE (6).

**Table 3:** SERPING1 genetic testing result of the proband.

Gene	Transcript/variant	Protein change	Zygosity	Classification	Interpretation
SERPING1	NM_000062.3:c.1480C>T	p.Arg494Ter (p.R494*)	Heterozygous	Likely pathogenic	Nonsense variant supporting SERPING1-associated type 1 HAE-C1INH in the clinical and complement context

## Family investigation

**Figure 2:**
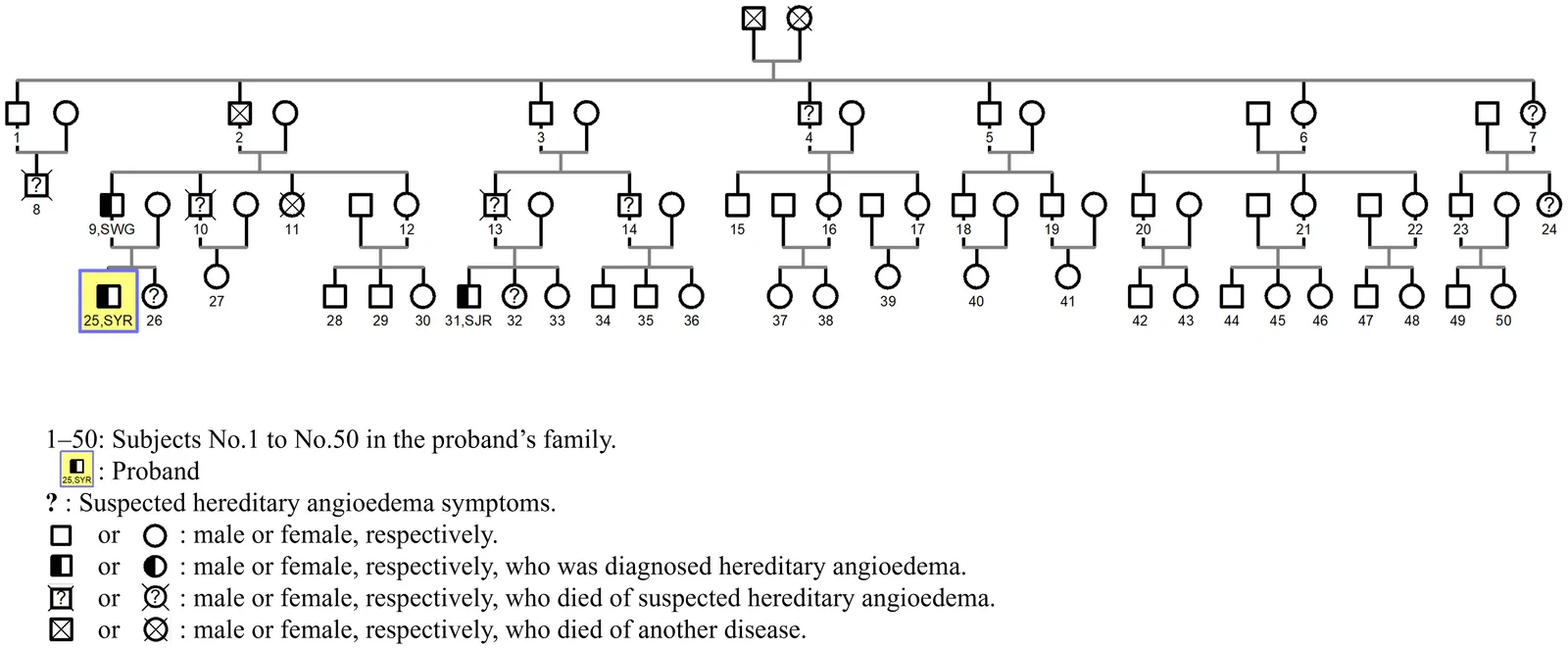
Family pedigree showing the proband and clinically suspected affected individuals across three generations. P1 indicates the proband; P2 indicates the proband’s father; P3 indicates the proband’s paternal second cousin. Filled symbols indicate laboratory-confirmed HAE-C1INH. Question-mark symbols indicate clinically suspected affected relatives based on family history. Asterisks indicate relatives who reportedly died from laryngeal edema.

After obtaining written informed consent, we collected family history information from 50 traceable relatives across three generations. For living relatives, information was obtained through structured interviews focused on recurrent, self-limited swelling, abdominal symptoms, laryngeal symptoms, age at onset, triggers, treatment, and outcome. Information on deceased individuals was obtained from family recollection and available records. The pedigree is shown in **Figure 2**.

Among the 50 traceable relatives, 13 individuals were classified as clinically suspected affected relatives based on recurrent self-limited swelling involving the extremities, genital region, or upper airway, or fatal laryngeal attacks reported by family members. Three of these clinically suspected affected relatives reportedly died from laryngeal edema between the ages of 20 and 40. Importantly, laboratory confirmation was available only for the proband and two relatives who attended our center; therefore, the remaining relatives were classified as clinically suspected rather than biochemically confirmed cases.

C1-INH antigen, functional C1-INH, and C4 were measured in three relatives who accompanied the patient to our center. Two of them fulfilled laboratory criteria for type-I HAE (**Table 2**). P2, the proband’s father, reported recurrent swelling of the hands, feet, and scrotum several times per year. P3, the proband’s paternal second cousin, was an 8-year-old boy whose predominant manifestation was recurrent abdominal pain. The newly diagnosed relatives were advised to seek immediate medical attention for any laryngeal symptoms and were counseled about the potentially life-threatening nature of upper-airway attacks. Cascade genetic testing was recommended but was not completed in relatives.

## Discussion

This case highlights the diagnostic difficulty of abdominal HAE-C1INH in gastroenterology practice. The patient’s recurrent abdominal pain, diarrhea, bowel wall edema, and transient ascites repeatedly suggested infectious gastroenteritis. However, several features were inconsistent with routine bacterial gastroenteritis or peritonitis: repeated self-limited attacks, absence of fever or gastrointestinal bleeding, negative stool and ascites studies, limited response to antibiotics, and abrupt resolution of ascites. Together, these features support bradykinin-mediated intestinal angioedema as the mechanism of the abdominal episodes.

Abdominal attacks in HAE may result from bradykinin-induced vascular leakage in the bowel wall and peritoneal cavity, producing transient bowel wall edema, pseudo-obstruction-like symptoms, and sterile ascites (4, 5). When abdominal symptoms dominate the clinical picture, patients may undergo repeated emergency evaluations, unnecessary antimicrobial therapy, or even avoidable surgery. This is particularly relevant for gastroenterologists because C4 measurement is inexpensive and widely available. A low C4 level during a compatible acute attack should be interpreted as an important screening clue, but not as a standalone diagnostic test. Confirmation requires C1-INH antigen and functional testing, and genetic analysis can provide additional support, especially in pedigrees or diagnostically uncertain cases (1).

The molecular result strengthened the diagnosis in the present case. The proband carried a heterozygous SERPING1 nonsense variant, NM_000062.3:c.1480C>T (p.Arg494Ter/R494*), classified as likely pathogenic. Nonsense variants can lead to premature termination of the C1-INH protein or loss of functional protein production, consistent with the low C1-INH antigen and function observed on repeat testing. The same variant has previously been reported among Chinese HAE patients (6), providing additional support for its disease relevance in the present clinical context.

The family investigation is clinically important but must be interpreted cautiously. The original pedigree suggested a large number of affected relatives and fatal laryngeal attacks, emphasizing the public health value of family counseling. However, most affected relatives in this family were identified on clinical grounds rather than by laboratory or genetic confirmation. Therefore, the revised manuscript deliberately distinguishes clinically suspected relatives from biochemically confirmed cases. This distinction is important because retrospective family recall may overestimate or underestimate true disease status, particularly for deceased relatives or for living relatives with mild or atypical symptoms.

Treatment and counseling should be individualized. On-demand therapies such as icatibant or plasma-derived C1-INH can be used for acute attacks, and long-term prophylaxis may be considered according to attack frequency, severity, airway risk, patient preference, and drug availability (1, 7, 8). In this patient, a single dose of icatibant was administered after the diagnosis in the setting of a recently resolving acute abdominal edema episode, and no recurrence was reported during follow-up. The family history of fatal laryngeal edema underscores the need for education about airway symptoms, emergency access to effective therapy, and genetic counseling.

This report has several limitations. First, laboratory confirmation was available only for the proband and two relatives, while most affected family members were classified as clinically suspected. Second, cascade genetic testing was not completed in relatives, so segregation of the SERPING1 variant within the pedigree could not be demonstrated. Third, some family history information, especially for deceased relatives, was based on retrospective recall. Fourth, the exact onset of the proband’s peripheral swelling could not be precisely dated. Fifth, complement and C1-INH testing was performed at different time points and in different laboratories using different units and reference ranges; however, both sets of results supported impaired C1-INH function and low C4. These limitations were acknowledged to avoid overinterpretation of the family screening results.

The patient expressed relief at finally receiving a definitive diagnosis after years of repeated hospitalizations and ineffective treatments. He stated, “I am grateful to understand what has been causing my symptoms all these years. Knowing that I have effective treatment available and that my family members can also be helped gives me peace of mind.” The patient emphasized the importance of educating healthcare providers about this rare condition to prevent similar diagnostic delays for others.

## Conclusion

HAE-C1INH should be considered in patients with recurrent, self-limited abdominal pain, transient ascites, bowel wall edema, and negative microbiological evaluation. Serum C4 is a practical screening test during compatible acute episodes, but diagnosis requires C1-INH antigen/function testing and, when available, genetic confirmation. Accurate diagnosis can prevent unnecessary interventions and enables counseling and targeted evaluation of at-risk relatives.

## Data Availability

The original contributions presented in the study are included in the article/**Supplementary Material**. Further inquiries can be directed to the corresponding authors.
