# Supplementary material for: Case Report: Hereditary angioedema masquerading as gastroenteritis
Source: Front Immunol. 2026 Aug 26;17:1828352. doi: 10.3389/fimmu.2026.1828352 (PMC13553354; doi:10.3389/fimmu.2026.1828352)
Supplement: Supplementary file 1 [file DataSheet1.pdf]

CARE Checklist (2013)

Information to include when writing a case report

Manuscript Title: Case Report: Hereditary Angioedema Presenting as Recurrent Abdominal Attacks Masquerading as Gastroenteritis

Journal: Frontiers in Immunology

Item	Checklist Item	Description	Reported
1	**Title**	The diagnosis or intervention of primary focus followed by the words "case report"	Yes
2	**Key Words**	2 to 5 key words that identify diagnoses or interventions in this case report, including "case report"	Yes
3a	**Abstract - Introduction**	What is unique about this case and what does it add to the scientific literature?	Yes
3b	**Abstract - Main Symptoms**	Main symptoms and/or important clinical findings	Yes
3c	**Abstract - Diagnoses**	The main diagnoses, therapeutic interventions, and outcomes	Yes
3d	**Abstract - Conclusion**	What is the main "take-away" lesson(s) from this case?	Yes
4	**Introduction**	One or two paragraphs summarizing why this case is unique (may include references)	Yes
5a	**Patient Information**	De-identified patient specific information	Yes
5b	**Primary Concerns**	Primary concerns and symptoms of the patient	Yes
5c	**Medical/Family History**	Medical, family, and psycho-social history including relevant genetic information	Yes
5d	**Past Interventions**	Relevant past interventions with outcomes	Yes
6	**Clinical Findings**	Describe significant physical examination (PE) and important clinical findings	Yes
7	**Timeline**	Historical and current information from this episode of care organized as a timeline	Yes
8a	**Diagnostic Testing**	Diagnostic testing (such as PE, laboratory testing, imaging, surveys)	Yes
8b	**Diagnostic Challenges**	Diagnostic challenges (such as access to testing, financial, or cultural)	Yes
8c	**Diagnosis**	Diagnosis (including other diagnoses considered)	Yes
8d	**Prognosis**	Prognosis (such as staging in oncology) where applicable	N/A
9a	**Types of Intervention**	Types of therapeutic intervention (such as pharmacologic, surgical, preventive, self-care)	Yes
9b	**Administration**	Administration of therapeutic intervention (such as dosage, strength, duration)	Yes
9c	**Changes**	Changes in therapeutic intervention (with rationale)	N/A

Item	Checklist Item	Description	Reported
**10a**	**Outcomes**	Clinician and patient-assessed outcomes (if available)	Yes
**10b**	**Follow-up Results**	Important follow-up diagnostic and other test results	Yes
**10c**	**Adherence**	Intervention adherence and tolerability (How was this assessed?)	Yes
**10d**	**Adverse Events**	Adverse and unanticipated events	None reported
**11a**	**Discussion - Strengths/Limitations**	A scientific discussion of the strengths AND limitations associated with this case report	Yes
**11b**	**Discussion - Literature**	Discussion of the relevant medical literature with references	Yes
**11c**	**Discussion - Rationale**	The scientific rationale for any conclusions (including assessment of possible causes)	Yes
**11d**	**Discussion - Take-away**	The primary "take-away" lessons of this case report (without references) in a one paragraph conclusion	Yes
**12**	**Patient Perspective**	The patient should share their perspective in one to two paragraphs on the treatment(s) they received	Yes
**13**	**Informed Consent**	Did the patient give informed consent? Please provide if requested	Yes

The CARE checklist is used to improve the completeness and transparency of case reports. Authors should address each item on the checklist and indicate where in the manuscript each item is reported.
